# Editorial: The role of macrophages in metabolic disorders

**DOI:** 10.3389/fphys.2023.1308625

**Published:** 2023-10-20

**Authors:** Yagna P. R. Jarajapu, Alyssa H. Hasty

**Affiliations:** ^1^ Department of Pharmaceutical Sciences, College of Health Professions, North Dakota State University, Fargo, ND, United States; ^2^ Department of Molecular Physiology and Biophysics, School of Medicine, Vanderbilt University, Nashville, TN, United States; ^3^ Tennessee Valley Healthcare System, US Department of Veterans Affairs, Nashville, TN, United States

**Keywords:** macrophages, inflammation, metabolsim, reprogramming, polarization

A chronic inflammatory environment increases risk for the development of a plethora of clinical conditions including atherosclerosis, cardiorenal disease, cognitive decline and dementia, frailty and cancer. Altered myelopoiesis and dysregulated activation or paracrine switch in monocyte-macrophages largely contribute to the pro-inflammatory environment in metabolic disorders (Barrett et al., 2017; Nagareddy et al., 2013).

Though long-known to carry out phagocytosis, macrophages are increasingly proven to play diverse physiological functions. Because of a high degree of heterogeneity and phenotypic plasticity, macrophages play a beneficial role in the tissue homeostasis. Macrophages are highly responsive to the environment, which dictates their pro- or anti-inflammatory phenotype, and participate in either regenerative or pathological processes (De Santa et al., 2019). Evidence is accumulating in support of the cellular metabolic state being a strong determinant of the functional phenotype of macrophages (Caslin et al., 2020). However, signaling pathways that facilitate metabolic switches leading to an altered functional states are yet to be delineated. Metabolic reprogramming to induce a phenotypic switch from pro-inflammatory to anti-inflammatory/regenerative macrophages is emerging as a new therapeutic approach for the treatment of chronic inflammatory disorders such as atherosclerosis, rheumatoid arthritis and complications associated with diabetes, obesity and aging (Peterson et al., 2018; Minhas et al., 2021). On the other hand, evidence is also accumulating in support of macrophages altering cellular metabolism of target cells leading to metabolic dysfunction and local or systemic pathology.

A series of articles published under the theme, *The Role of Macrophages in Metabolic Disorders*, mainly focus on different scenarios of how macrophages influence cellular metabolism of other cells, and how dysregulated macrophage metabolism disturbs homeostasis in the effector organ. Mouton et al. provided an extensive review of the reparative functions of macrophages driven by metabolism of different fuels in cardiorenal diseases. Lactate accumulation followed by cardiac and renal injury may activate a macrophage reparative phenotype; however, the review identifies a gap in the knowledge with respect to the role of lactate metabolism in driving macrophage polarization in different states. This review highlighted the differential role of HIF isoforms on macrophage metabolism of glucose and fatty acids. The authors presented evidence for the anti-inflammatory effects of ketones in macrophages via metabolic and nonmetabolic pathways and highlighted the pathological significance of impaired ketone metabolism in the diabetic myocardium. They hypothesized that ketone supplementation might be a promising approach for restoring mitochondrial metabolism in diabetes. Finally, a differential role of macrophages in the reparative fibrosis in acute and chronic injury conditions was briefly reviewed.

Callegari et al. reviewed evidence in support of physical exercise as an intervention to combat systemic inflammation and to restore physiological homeostasis in subjects with or without metabolic disorders. The authors elegantly reviewed several lines of evidence that showed beneficial effects of either long-term or a single-bout exercise programs in reversing inflammation in individuals with metabolic disorders by skewing macrophage polarization towards an anti-inflammatory phenotype in conditions such as insulin resistance, atherosclerosis, non-alcoholic hepatic steatosis, non-alcoholic fatty liver disease, and tumor microenvironment. This review also identified selected signaling pathways that are activated by exercise that can be targeted to ameliorate systemic inflammation. Notably, this review presented no evidence for the reversal of myelopoietic bias in hematopoietic stem cells in metabolic disorders by exercise, which requires systematic investigation.

The review by Li et al. provide a critical appraisal of heterogeneity of macrophage populations based Ly6C expression in different scenarios such as homeostasis, autoimmune disease, tissue fibrosis and cancer. Origin, maturity, and functions of different subpopulations of macrophages were described. Interestingly this review quoted the existence and function of Ly6C-intermediate (Ly6C^int^) population. The accompanying original research article by Zhang et al. takes the heterogeneity of macrophages to another level of understanding by delineating subpopulations of anti-inflammatory macrophages in placental decidua and chorionic villi derived from healthy or type 2 diabetic subjects. Authors report localizing M1, M2a, M2b and M2c subpopulations of macrophages in these tissues with M2a as the predominant population. While the findings are novel, the study is observational and was carried out in a few numbers of Asian subjects.

Lastly, an interesting review by Kang et al. described how platelet-macrophage interactions shape the tumorigenic microenvironment. This review summarized findings that showed evidence for the metabolic dysregulation in macrophages induced by platelet-derived exosomes and switching immunosuppressive phenotype to pro-inflammatory tumor-promoting phenotype. This review highlighted the potential of metabolic reprogramming in tumor-associated macrophages for promoting the anti-inflammatory and anti-tumorigenic potential.

Collectively, this series of articles provides up-to-date information on macrophage metabolism and heterogeneity in metabolic diseases, and sheds light on to the approaches of metabolic reprogramming for ameliorating systemic inflammation associated with metabolic disorders.
